# Efficacy of Fluoride Varnishes with Added Calcium Phosphate in the Protection of the Structural and Mechanical Properties of Enamel

**DOI:** 10.1155/2017/7834905

**Published:** 2017-12-07

**Authors:** Mahdi Shahmoradi, Neil Hunter, Michael Swain

**Affiliations:** ^1^Department of Dental Biomaterials, The University of Sydney, Sydney, NSW, Australia; ^2^Institute of Dental Research, Westmead Centre for Oral Health and The Westmead Institute for Medical Research, The University of Sydney, Westmead, NSW, Australia

## Abstract

The aim of this study was to investigate the efficacy of various fluoride varnishes in the protection of the structural and nanomechanical properties of dental enamel. Demineralized enamel specimens were imaged using a high-resolution micro-CT system and lesion parameters including mineral density and lesion depth were extracted from mineral density profiles. Nanoindentation elastic modulus and hardness were calculated as a function of penetration depth from the load-displacement curves. The average depth of the lesion in specimens with no prior fluoride varnish treatment was 86 ± 7.19 *μ*m whereas the varnish treated specimens had an average depth of 67 ± 7.03 *μ*m (*P* < 0.05). The mineral density of enamel lesions with no fluoride varnish treatment had an average of 1.85 gr/cm3 which was 25% lower than the corresponding value in varnish treated enamel and 37% lower than sound enamel. While, in the varnish treated group, elastic modulus and hardness values had decreased by 18% and 23%, respectively, the corresponding values in the non-varnish treated specimens had a reduction of 43% and 54% compared to the sound enamel. The findings from this study highlight the preventive role of fluoride varnishes. Addition of calcium and phosphate does not seem to enhance or inhibit the prevention or remineralization performance of fluoride varnishes.

## 1. Introduction

Dental caries, as one of the most common chronic diseases, is the result of the disruption of the microbial homeostasis of dental plaque and the consequent breakdown of the mineral equilibrium between enamel and the biofilm fluid. In recent decades, improved understanding of the ultrastructure and the dynamic nature of enamel caries [1] has led to a paradigm shift in the management of lesions from an operative-restorative approach towards a prevention-remineralization concept. The objective of contemporary caries management is to shift the dynamic balance of the demineralization-remineralization reactions towards net mineral gain within the lesion, through the provision of bioavailable mineralizing ions. Fluoride has been proven to play a key role in this process [2, 3] and, therefore, it has been employed widely through various vehicles such as water fluoridation, fluoridated toothpastes, and mouthwashes [4–7].

Endeavoring to prolong the bioavailability and the retention of fluoride onto the tooth surface, Schmidt proposed a method for carrying sodium fluoride using a natural colophony base which could adhere to the tooth surface in the presence of saliva [8, 9]. Current formulations of fluoride varnishes mostly include 5% sodium fluoride (22,600 ppm) and can be applied to all tooth surfaces twice per year or as a spot application when necessary. Besides easy application and high patient acceptability, fluoride varnishes have exhibited significant clinical efficacy in the reduction of dental caries [10, 11]. It is suggested that the highly concentrated fluoride in varnish binds to the surfaces of the enamel hydroxyapatite crystals and forms calcium fluoride [12]. These precipitated globules of calcium fluoride act as a reservoir and gradually release fluoride during future acid attacks. The released fluoride prevents demineralization of enamel and reduces caries susceptibility through several suggested mechanisms. These include the incorporation of fluoride into enamel hydroxyapatite via filling or displacing the hydroxyl vacancies, therefore stabilizing the crystal structure and lowering the solubility product (Ksp) [12].

Although essential, fluoride alone is not sufficient for the prevention of demineralization and possible redeposition of minerals into hydroxyapatite crystals. The presence of bioavailable calcium and phosphate in the plaque fluid is also necessary for maintaining the structural integrity of hydroxyapatite crystals. While normal saliva does not usually contain an optimal level of fluoride, it has been shown that saliva is supersaturated with respect to calcium and phosphate [13]. Several proteins including statherins are responsible for stabilizing calcium and phosphate in saliva and preventing their spontaneous precipitation [14]. However, with the aim of increasing the amount of calcium and phosphate in saliva and desiring to improve the demineralization prevention efficacy and possible remineralization of the lesion, fluoride varnishes with added calcium and phosphate compositions have been introduced recently. These include fluoride varnishes with added amorphous calcium phosphate (Enamel Pro® Varnish), tri-calcium phosphate (Clinpro White varnish™), casein phosphopeptide stabilized amorphous calcium phosphate (MI Varnish™ with RECALDENT™), and Xylitol coated calcium and phosphate (Embrace Varnish). Considering the recent development of these formulations for sodium fluoride varnishes, the effect of the addition of calcium and phosphate in amplifying or debilitating the action of fluoride remains unclear. Therefore the aim of this study was to investigate the efficacy of various fluoride varnishes in the protection of the structural and nanomechanical properties of dental enamel and to examine the effect of added calcium and phosphate on the performance of different fluoride varnishes in vitro.

## 2. Materials and Methods

### 2.1. Study Specimens

Extracted human premolars were collected from the Oral Surgery and Orthodontics departments at Sydney Dental Hospital, University of Sydney, according to protocols approved by Sydney local health district ethics review committee, protocol numbers X12-0065 & HREC/12/RPAH/106. Extracted teeth were cleaned, disinfected, and visually assessed by a clinician and thirty teeth with no lesions or restorations were selected. Consequently, each caries free tooth was derooted and sectioned vertically through the crown to provide two or three specimens resulting in sixty specimens in total. All of the cut surfaces plus one-third of the enamel surface (as a reference) were covered with an acid-resistant nail varnish.

### 2.2. Varnish Application and Initial Caries Development

Enamel specimens were randomly allocated to five different groups (*n* = 12 and *N* = 60) for demineralization and pH-cycling treatments based on the type of varnish as below:

  Group 1: Control, no application of varnish
  Group 2: Clinpro™ White Varnish
  Group 3: Duraphat® fluoride varnish
  Group 4: MI Varnish
  Group 5: Duraphat Single Dose fluoride varnish

Details of the commercial fluoride varnishes including the list of their constituent agents are provided in Table 1. For each group of materials, a thin layer of fluoride varnish was applied on half of the enamel surface according to the product instruction. Gentle air pressure was used to remove any excess varnish. The samples were then immersed in artificial saliva (30 mL per sample) for 4–6 hours based on the varnish manufacturer's instruction. The artificial saliva used was a pH 6.9 solution prepared using 0.2 mM glucose, 9.9 mM NaCl, 1.5 mM CaCl_2_·2H_2_O, 3 mM NH_4_Cl, 17 mM KCl, 2 mM NaSCN, 2.4 mM K_2_HPO_4_, 3.3 mM urea, and 2.4 mM NaH_2_PO_4_ [15]. A thin blade was used to remove the set varnish from the surface of the tooth. Acetone wipe was used to remove any remaining varnish residue. Each specimen was then immersed in 50 ml of demineralization solution for 21 days (37°C) to undergo demineralization and to form an initial subsurface carious lesion. Demineralization solution was a pH 4.5 lactic acid (0/1 M/l) solution, containing 6% w/v methylcellulose and 500 mg/l hydroxyapatite prepared under vigorous stirring, and was similar to the demineralization solution used by other researchers [16]. Specimens were then subjected to three weeks of pH-cycling during which the enamel samples were immersed for 4 hours in a demineralizing solution (0.05 M acetate buffer, pH 5.0 and containing 1.28 mM Ca, 0.74 mM P, and 0.03 *μ*g F/mL) and for 20 hours in artificial saliva (150 mM KCl, 1.5 mM Ca, 0.9 mM P, 0.05 *μ*g F/mL in 0.1 M Tris buffer, pH 7.0) each day. The demineralization solution and artificial saliva were replenished with fresh solutions every two days and the jars containing teeth were stored at a temperature of 37°C. The researchers were blinded regarding group allocation which was performed by an assistant.

### 2.3. Mineral Density Characterization of Lesions after Acid Attack

Mineral density distributions of enamel specimens after the application of various varnishes, demineralization, and pH-cycling treatment were characterized using high-resolution desktop microcomputed tomography system (Skyscan 1172, Skyscan N.V, Aartselaar, Belgium) at an accelerating source voltage of 100 keV, a source current of 100 *μ*A, and an exposure time of 885. Grey level calibration was achieved using three hydroxyapatite discs with low, medium, and high mineral density in order to determine the mineral density values of different parts of the tooth. Details of the employed phantoms are provided elsewhere [17].

Low energy X-rays were eliminated using an inbuilt filter equal to 1.0 mm thickness of aluminum and 0.05 mm of copper to restrict the spectral bandwidth of the polychromatic radiation. The equivalent monochromatic energy spectrum of filtered X-ray had an effective mean energy of 60 keV. The long axes of the teeth were parallel with the center of rotation of the mounting device. During the scanning process, the samples were rotated over 360° at angular increments of 0.14° generating 2570 two-dimensional shadow projections with an image matrix of 2000 pixels  ×  1048 pixels. These images were saved as 16-bit Tagged Image File Format (TIFF) and consequently exported to a 3D cone beam reconstruction program (NRecon software, version 1.4.4; SkyScan) for the tomographic reconstruction of the 3-D object and production of tomographic images. The tomographic reconstruction produced a dataset of slice views in 16 bit TIFF format, which were perpendicular to the specimen rotation axis and had a voxel size resolution of 8–10 *µ*m.

### 2.4. Visualization and Quantification of Demineralization

The produced tomographic images were consequently imported into MATLAB (MatLab R2012b 8.0.0.783, Mathworks, Natick, MA, USA) for the denoising process and for increasing the signal to noise ratio of micro-CT images. Denoising was performed using a method based on total variation regularization [18, 19], with the following parameters:

 
*μ* (regularization parameter) = 0/04.
 
*ρr* (initial penalty parameter) = 3.
 
*ρo* = 40.

Visualization and color-coding of the lesions were performed using the colormapeditor command in MATLAB by choosing Jet color map with fixed RGB (Red, Green, Blue) index values for all of the colorized images. The color codes were based on the grey level values and corresponding mineral densities of each specimen, producing calibrated mineral maps. The calibration of mineral density was implemented by measuring and averaging grey level values of 10 points on selected images of each hydroxyapatite phantom, followed by plotting the obtained grey level values against the mineral density value of that phantom. Based on plotted values, the calibration equation was calculated and used to transform grey level values of the images into true mineral density values.

Analysis and quantification of the parameters including mineral density and the depth of the lesion were performed using mineral density profiles plotted in FIJI (W.S. Rasband, U. S. National Institutes of Health, Bethesda, MD, USA) and the visualized mineral maps in MATLAB, on a computer system with Intel® Core™ i7 CPU (Q740 @ 1.73 GHz), and 4.00 GB RAM memory. For calculating the mineral density of each region (ROI), values of all pixels in the specific region were measured and averaged. The depth of the carious lesions was measured using line scans of mineral content profile across the surface layer of the lesions.

### 2.5. Nanoindentation Mechanical Characterization

Fifteen specimens were selected and sectioned horizontally for nanoindentation mechanical testing to measure hardness and elastic modulus values across the lesions. Each specimen was embedded in a cold-curing epoxy resin (Epofix, Struers, Denmark). Upon curing the occlusal surface was ground flat with silicon carbide papers with grit sizes 240, 400, 600, and 1200 under continuous water irrigation to avoid overheating. Specimens were then polished with 9 *μ*m and 1 *μ*m diamond polishing pastes and finally with 0.04 *μ*m colloidal silica. After each polishing cycle, specimens were ultrasonically cleaned (Unisonics, Australia) for 5 min and stored in Hank's balanced salt solution HBSS (Sigma–Aldrich, Germany) at room temperature.

**Figure 1:**
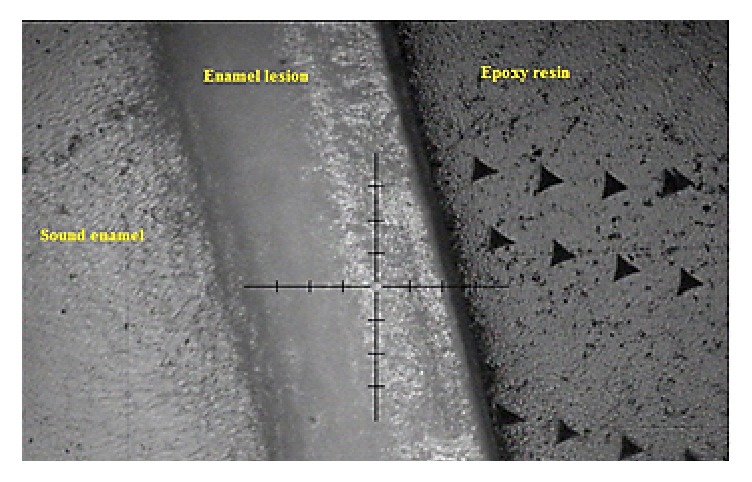
Stereomicroscopic image showing the indentation marks through the artificial enamel lesion.

The indentation experiment was conducted using an Ultra Micro Indentation System (UMIS-2000, CSIRO, Australia), equipped with a three-sided Berkovich indenter tip calibrated on a fused silica standard sample of known properties. Specimens (*n* = 15) were nanoindented using a maximum load of 20 mN. A total of 10 indents separated from each other by 15 *μ*m were positioned across the lesion in each specimen (Figure 1). The UMIS system software (Ibis, Fisher-Cripps laboratories, Australia) was used for the subsequent calculation of the elastic modulus and hardness of the samples tested. The nanoindentation hardness (*H*) is the contact pressure of the indenter divided by the projected contact area (*A*) of the sample at maximum load (*P*_max_) which can be estimated from [20]

(1) $$\begin{array}{c} H=\frac{P_{max}}{A}, \end{array}$$

whereas elastic modulus of the specimen *E*_*s*_ was calculated from [20]

(2) $$\begin{array}{c} \frac{1}{E_{r}}=\frac{\left(1-{v_{s}}^{2}\right)}{E_{s}}+\frac{\left(1-{v_{i}}^{2}\right)}{E_{i}}, \end{array}$$

where *E*_*r*_ is the indentation elastic modulus (reduced modulus) and *E*_*i*_ and *v*_*i*_ are the elastic modulus and Poisson's ratio of the diamond indenter, 1070 GPa and 0.07, respectively. Poisson's ratio, *v*_*s*_, for enamel is 0.3. The data were analyzed according to Oliver and Pharr [20] and the values of hardness and elastic modulus were averaged from the results of 10 indents.

### 2.6. Statistical Analysis

The measurements were checked for normal distribution. Statistical analysis was performed using statistical software GraphPad Prism (GraphPad Software. San Diego, CA) to test for differences between the means. The results of mineral density quantification and nanoindentation mechanical testing in different groups were analyzed by One-way analysis of variance (ANOVA). Multiple comparisons between groups were performed by post hoc Tukey test. *P* values less than 0.05 were considered to be statistically significant.

## 3. Results

### 3.1. X-Ray Micro-CT Mineral Density Characterization

**Figure 2:**
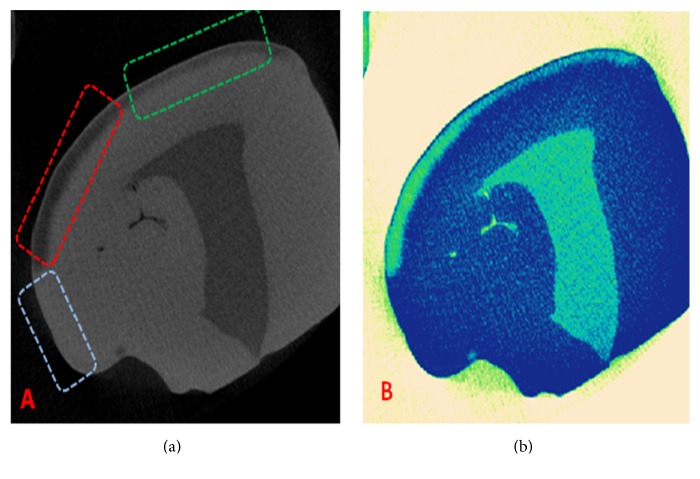
(a) Nominated micro-CT image of an artificial lesion after three weeks of acid attack showing the demineralization prevention effect of fluoride varnish in the green rectangular area compared to the non-varnish treated control area (shown by the red rectangle) and the protected sound enamel (shown by the blue rectangle). (b) Mineral map of the lesion in Figure 2(a).

Nominated micro-CT and color-coded images of the enamel lesion after varnish application, initial acid attack, and three weeks of pH-cycling are presented in Figure 2. The visualized and color-coded images are normalized mineral density maps which show the distribution and mineral density levels within the lesion and sound enamel areas.

Regarding the evaluation of demineralization prevention of different fluoride varnishes, micro-CT results indicated that the application of all fluoride varnishes significantly protected the enamel against acid attack and decreased the progression of the lesion when compared to the control group. Accordingly, the average depth of the lesion in artificial lesions with no prior fluoride varnish treatment was 86 ± 7.19 *μ*m whereas the varnish treated sections had an average depth of 67 ± 7.03 *μ*m. The mineral density of sound enamel ranged from 2.43 to 2.89 gr/cm^3^. The mineral density of the lesion sections with no fluoride varnish treatment had a mean value of 1.85 gr/cm^3^, which was 25% lower than the corresponding value in varnish treated sections and 37% lower than sound enamel (*P* < 0.05).

Comparison of the depth and mineral density of the lesions treated with different varnish types indicated no significant difference in depth reduction and mineral density preservation among different groups including conventional fluoride varnishes and fluoride varnishes with added calcium and phosphate (*P* > 0.05).  Table 2 shows the mean ± SD value of lesion depth reduction (*μ*m) and demineralization reduction (g/cm^3^) for different fluoride varnishes and control groups.

### 3.2. Nanoindentation Mechanical Testing

**Figure 3:**
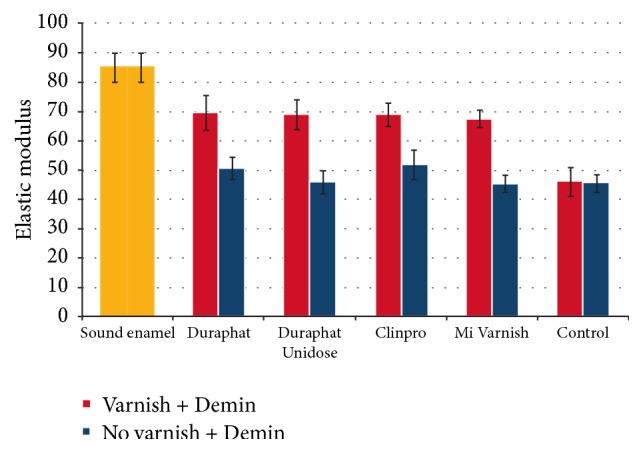
Nanoindentation elastic modulus of sound, control, and study groups in varnish (Red) and non-varnish treated (blue) enamel after varnish application, demineralization, and pH-cycling treatments.

Nanoindentation elastic modulus and hardness were calculated as a function of penetration depth from the load-displacement curves.  Figure 3 shows the average and standard deviations of the elastic modulus of enamel in control, varnish treated lesions, and sound enamel. Mechanical properties of the sound enamel which were measured to serve as the reference value showed a range of 73 to 108 GPa for the elastic modulus and a range of 4.2 to 6.6 GPa for the cross-sectional hardness. While, in the varnish treated demineralized enamel, elastic modulus and hardness values had decreased 18% and 23%, respectively, the corresponding values in the non-varnish treated specimens had a reduction of 43% and 54% compared to the sound enamel (Figure 3). There was no significant difference in the amount of hardness and elastic modulus reduction of enamel among different varnish groups (*P* > 0.05).

## 4. Discussion

Different models have been developed for the study of demineralization and remineralization processes including in situ models, pH-cycling models, and in vitro models using acidic gel or solution systems [21, 22]. In the current study, we employed a model comprised of an initial subsurface lesion followed by three weeks of pH-cycling as it accurately simulates the in vivo cyclic episodes of demineralization and remineralization. Although the depth of the created artificial lesions is generally lower and the surface layer is thinner than natural lesions, the resultant lesions exhibit a standard depth and high reproducibility under controlled conditions and therefore minimized the effect of substrate variability on experimental results.

In our research, we studied the cross-sectional properties of acid challenged enamel pretreated with various fluoride varnishes through the characterization of two sets of different properties, the mechanical properties (hardness and elastic modulus) and the mineral properties (mineral density and the depth of the lesion). For the evaluation of mineral change in the lesions, we used micro-CT which is capable of characterizing the mineral density and structural changes of the lesion without the need for sectioning or sample preparation [23]. The application of micro-CT for the characterization of mineral density of enamel lesions has been validated in previous studies [24, 25]. In addition, nanoindentation mechanical testing performed on the cross-section of the lesions, can evaluate the effect of mineral change on the mechanical property of the lesion in the subsurface area and it provides more reliable information compared to surface analysis methods using SEM or microhardness testing, which are generally performed on the external enamel surface [26]. The results of cross-sectional nanoindentation mechanical testing were shown to have a high correlation with mineral density of the tested material [27, 28].

The resultant mineral profiles obtained by X-ray microcomputed tomography and the results of the nanoindentation mechanical testing showed that, for all varnish types, varnish treated enamel had higher mineral density, lower lesion depth, higher hardness, and higher elastic modulus. These findings indicate that studied dental varnishes significantly protect enamel against acid attack and prevent the loss of its structural and mechanical integrity. However, none of the investigated varnishes could totally prevent or reverse the mineral loss by the lesion.

The higher mineral density and mechanical properties of varnish treated enamel can be attributed to the greater formation of calcium fluoride adsorbed on the surface of enamel and the role of fluoride in the inhibition of mineral loss in enamel. It is suggested that incorporation of fluoride in free spaces on the crystal lattice of the enamel apatite renders the fluoridated crystals (fluorapatite or hydroxyfluorapatite) more difficult to dissolve and easier to repair. In addition, fluoride acts as a catalyst and increases the reaction rate of mineral formation and transformation [29]. The protective role of fluoride has been demonstrated in various clinical studies [30]. However, to the knowledge of the authors, this is the first study to evaluate the effect of added calcium phosphate on the performance of varnishes using high-resolution methods including micro-CT and nanoindentation.

Based on the information from Material Safety Data Sheets (MSDS) of the studied varnishes, they are all basically composed of an active component (5% Sodium fluoride with or without added calcium phosphate) embedded in a mixture of excipient constituents. The role of the excipient components is to carry the active agent and to provide retention for the active ingredient through bonding to the enamel surface. Alcoholic solutions of resins are the main excipient components of most of the current varnishes. Alcohols are used as solvents to provide fluidity for the synthetic or natural resins (such as colophony, mastic, and shellac) used in the varnish materials. Following the application and exposure of the fluoride varnish to air, alcohols evaporate from the solution allowing the varnish to adhere to enamel surfaces for increased length of fluoride exposure [31]. Other excipient agents may be added by each company to promote features such as adhesion, shelf life, viscosity, handling, color, sweetness, and flavor of the material. A constituent found only in MI Varnish is silicon dioxide which is commonly used as a carrier of flavors and fragrances and also can function as a viscosity control agent, emulsion stabilizer, and a suspension and dispersion agent [32].

An unwanted possible risk of adding calcium and phosphate salts to fluoride ions in the varnishes is the formation of poorly soluble calcium fluoride phosphate phases [33] in the material package during storage or in the saliva after the application of the material. The formation of poorly soluble calcium fluoride phosphate phases either in the package or in the saliva can lead to the decrease in the number of bioavailable fluoride ions which are necessary for the formation of calcium fluoride globules on the tooth surface. Several strategies have been utilized to stabilize the fluoride, calcium, and phosphate ions in varnishes and prevent the formation of poorly soluble calcium fluoride phosphate phases during material storage. These include the coating of calcium and phosphate with Xylitol (in XCP™) [34], the stabilization of calcium and phosphate by casein phosphoprotein which is suggested to bind to amorphous calcium phosphate and to prevent the growth of calcium and phosphate ions to the critical size for nucleation and phase transformation [35] (in MI Varnish), and the prevention of the reaction between calcium and fluoride ions by a protective fumaric acid barrier which breaks and releases the calcium and phosphate ions when it comes into contact with saliva (in Clinpro). Although, theoretically, these stabilization methods may prevent the undesired reaction of fluoride with calcium and phosphate, given the supersaturation of calcium and phosphate within saliva, the addition of calcium and phosphate compositions to fluoride varnishes seems to be of limited benefit. The lack of difference in the efficacy of varnishes with added calcium phosphate composition and conventional varnishes observed in this study could be attributed to the considerable release of calcium and phosphate from the enamel substrate which happens during cycles of enamel demineralization and can potentially level off any difference in the amount of calcium and phosphate content among different groups.

In conclusion, the findings from the study of the mineral content and mechanical properties of demineralized enamel specimens in both study and control groups indicated that the application of the fluoride varnishes significantly decreased the progression of the lesion and inhibited the demineralization rate of enamel. These findings highlight the preventive role of fluoride varnish in the preservation of mineral structure and mechanical integrity of enamel against acid-induced demineralization. Addition of calcium and phosphate compositions does not seem to enhance or inhibit the performance of fluoride varnishes.

## Figures and Tables

**Table 1:** Details of fluoride varnishes used in the study. Duraphat and Duraphat Single Dose are representatives of conventional fluoride varnishes and MI Varnish and Clinpro are representatives of fluoride varnishes with added calcium phosphate compositions.

Product	Manufacturer	Active ingredient	Excipient ingredients	Source
Duraphat	Colgate Oral Care, Manufactured in Germany	5% Sodium fluoride (2.26% or 22,600 ppm of the fluoride ion)	Ethanol, White beeswax, Shellac, Colophony BP, Mastic, Sodium Saccharin, Flavor	MSDS
MI Varnish	GC Corporation, Itabashi-Ku, Tokyo, Japan	5% Sodium fluoride (2.26% or 22,600 ppm of the fluoride ion), Casein phosphopeptide-amorphous calcium phosphate (CPP-ACP)	Polyvinyl acetate (synthetic resin), Ethanol, Hydrogenated rosin, 1–5% Silicon dioxide, Flavor	MSDS
Duraphat Single Dose	Colgate-Palmolive Manufacturing, USA	5% Sodium fluoride (2.26% or 22,600 ppm of the fluoride ion)	Hydrogenated rosin resins, Ethanol, Benzyl Alcohol, Flavor	MSDS and manufacturer
Clinpro White Varnish	3M ESPE, St Paul, MN, USA	5% Sodium fluoride (2.26% or 22,600 ppm of the fluoride ion), Tri-calcium phosphate (TCP)	White modified rosin (Pentaerythritol glycerol ester of colophony resin), Ethyl alcohol, Water, xylitol, Flavor	MSDS

**Table 2:** Mean ± SD value of lesion depth reduction (*μ*m) and demineralization reduction (g/cm^3^) for different fluoride varnishes and control group.

Varnish type	Lesion depth reduction	Lesion density preservation
Duraphat	19.63 ± 4.07 *μ*m	0.58 ± 0.39 g/cm^3^
MI Varnish	18.50 ± 2.39 *μ*m	0.71 ± 0.32 g/cm^3^
Duraphat Single Dose	17.11 ± 5.33 *μ*m	0.69 ± 0.38 g/cm^3^
Clinpro White Varnish	19.74 ± 4.51 *μ*m	0.54 ± 0.25 g/cm^3^
Control	2.52 ± 1.79 *μ*m	0.09 ± 0.08 g/cm^3^
